# Distal embolization versus combined embolization techniques for blunt splenic injuries: comparison of the efficacy and complications

**DOI:** 10.18632/oncotarget.21527

**Published:** 2017-10-05

**Authors:** Yon-Cheong Wong, Cheng-Hsien Wu, Li-Jen Wang, Huan-Wu Chen, Kuo-Ching Yuan, Being-Chuan Lin, Yu-Pao Hsu, Shih-Ching Kang

**Affiliations:** ^1^ Emergency and Critical Care Radiology, Department of Medical Imaging and Intervention, Chang Gung Memorial Hospital, Linkou, Chang Gung University, Taoyuan City, Taiwan; ^2^ Division of Trauma and Emergency Surgery, Department of Surgery, Chang Gung Memorial Hospital, Linkou, Chang Gung University, Taoyuan City, Taiwan

**Keywords:** embolization technique, splenic injuries, efficacy, spleen infarct, splenic abscess

## Abstract

Comparable failure rates of distal or proximal transcatheter arterial embolization (TAE) techniques for blunt splenic injuries have been reported. This study is to investigate the efficacy and complication of combining both TAE techniques.

We included 26 patients of blunt splenic injuries for TAE therapy and randomized them into distal TAE and combined TAE groups. A prospective study was performed to compare their demographics, clinical parameters, hemograms, post-TAE splenic infarct volumes, splenic abscess and pancreatitis between the two groups.

Of 26 patients, 17 received distal TAE, 9 received combined TAE. Their basic demographics, clinical parameters and hemograms did not differ. Mean systolic blood pressure of all patients was significantly elevated after TAE at 24 hours later. Three patients of distal TAE group had residual pseudoaneurysms in follow up. They were considered failures in distal TAE group as opposed to all successes in combined TAE group. The risk difference of failure of distal TAE was 17.6%. None developed post-TAE splenic abscess, massive splenic infarct or pancreatitis. The mean splenic infarct volume of distal TAE (10.9%) versus combined TAE groups (6.6%) was not significant (*p* = 0.481).

Combined TAE is effective and safe to decrease the failure rates of non-operative management for blunt splenic injuries.

## INTRODUCTION

Non-operative management has become the standard of practice for blunt splenic injuries in hemodynamically stable patients [1–4]. About 20% of the non-operative management for splenic injuries might fail due to ongoing hemorrhage as a result of vascular injury and disruption of splenic capsule [1, 5]. Most vascular injuries can be depicted on multiphasic contrast-enhanced CT as contrast medium extravasation or pseudoaneurysm [4]. They are life-threatening and warrant immediate surgical treatment [6, 7].

If patients are responsive to fluid resuscitation, transcatheter arterial embolization (TAE) is a treatment of choice to improve splenic salvage rates [8–13]. Proximal TAE which is a technique of main splenic artery occlusion is adequate to stop splenic hemorrhage in most blunt splenic injuries [5, 10]. However, persistent pseudoaneurysms or new pseudoaneurysms could occur in about 10% of major injuries despite an initially successful proximal TAE [14–16]. This failure of proximal TAE can be attributed to rich collateral arteries distal to the site of main splenic artery occlusion [5, 10, 14, 16]. In these scenarios, distal TAE might be more effective whereby the injured branch arteries are superselectively occluded [5]. Nevertheless, distal TAE has been reported to associate with a greater risk of post-TAE events such as post-TAE infarct and delayed hemorrhage [8, 12]. Recent meta-analysis study has shown that failure rates in both techniques are comparable [5].

Our hypothesis for delayed splenic hemorrhage after distal TAE is the reopening of the spastic vessels at the non-embolized parts of the injured spleen, and this event could have been prevented should an additional proximal TAE was performed after a successful distal TAE. But to date, there is a limited study on combined TAE technique (combination of distal and proximal TAE techniques) as well as the potential complication it may cause such as spleen infarct [17]. Therefore in this study, we compare the efficacy and TAE-related complications between combined TAE and distal TAE techniques for blunt splenic injuries.

## RESULTS

**Figure 1:**
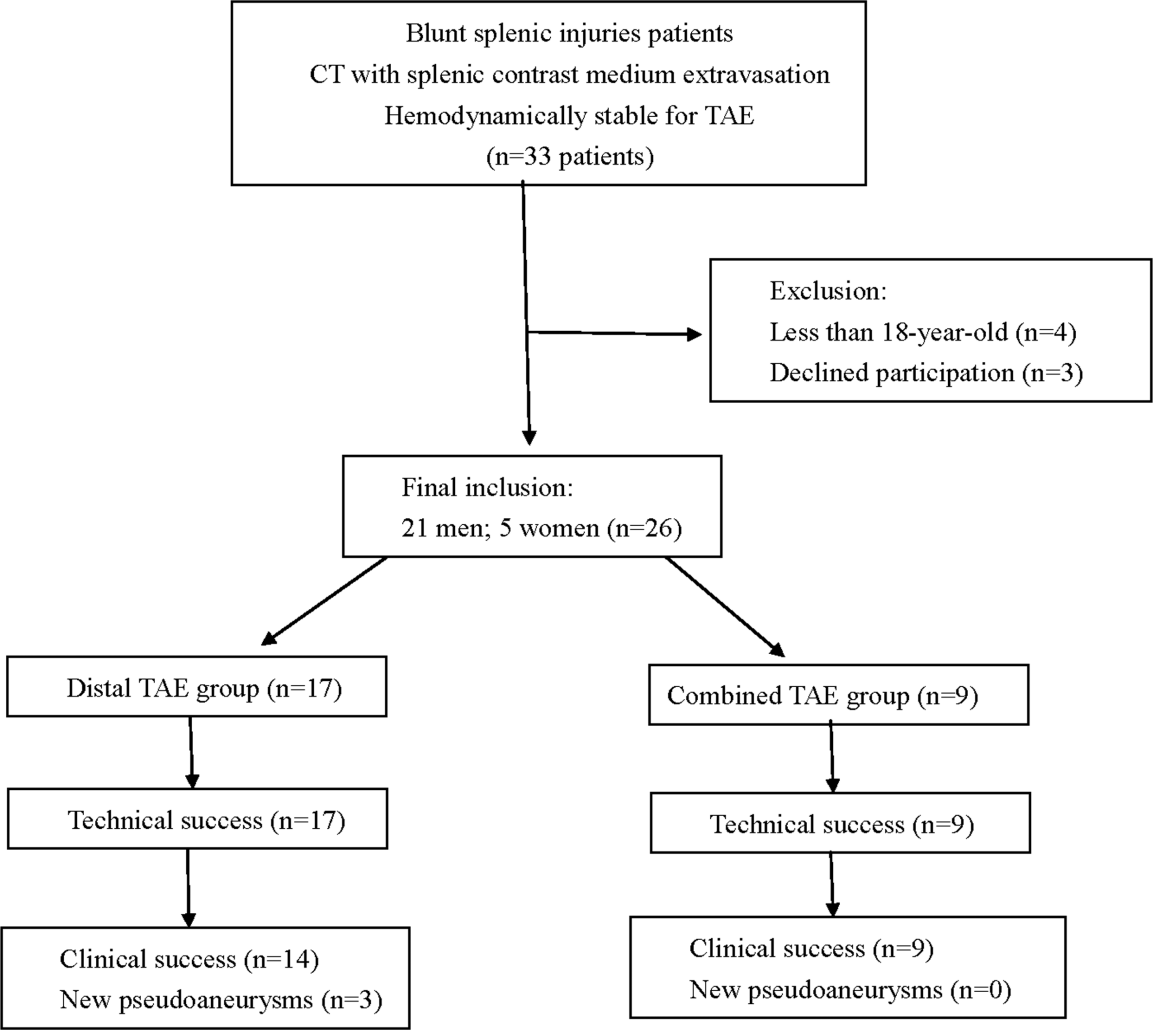
Flowchart of splenic TAE study

Within a two year study period, a total of 33 patients met the inclusion criteria. They were of blunt splenic injuries with CT findings of contrast medium extravasation and were hemodynamically stable for TAE treatment. We excluded four patients who were less than 18 year-old and three patients who declined to participate in this study (Figure 1). A total of 26 patients (21 men and 5 women) with a mean age of 39.8 ± 18.0 years (range, 18–74 years) were finally included. The mechanisms of injury were motor vehicle accident (*n* = 22), pedestrian versus car (*n* = 2) and falling from height (*n* = 2).

### Comparison of technical success and clinical success between distal TAE group and combined TAE group

**Figure 2:**
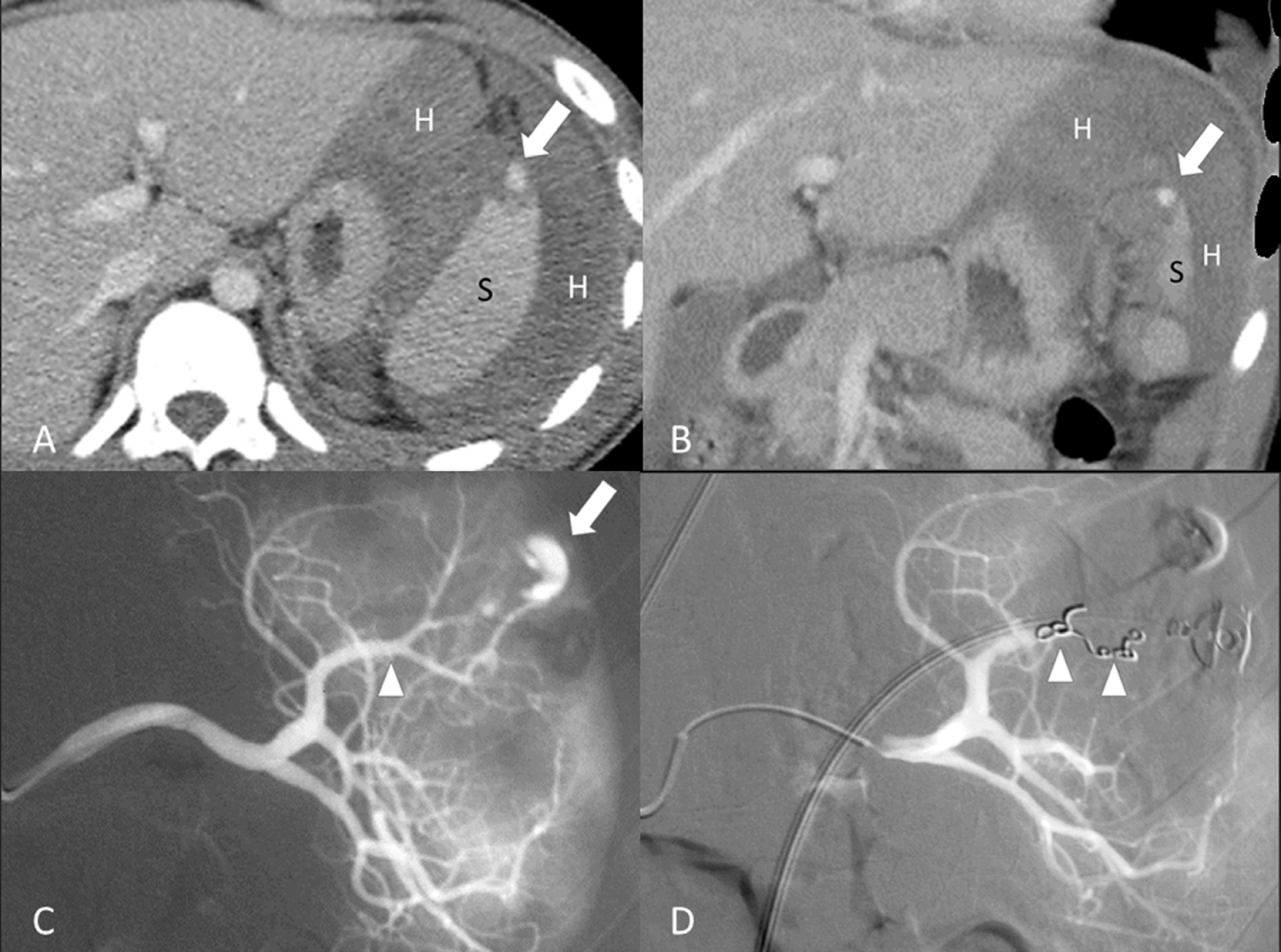
18-year-old male of grade II splenic injury underwent distal transcatheter arterial embolization (**A**, **B**) Contrast-enhanced axial and coronal CT at portal venous phase shows a pseudoaneurysm (arrow) at the lacerated spleen (S) surrounded by hemoperitoneum (H). (**C**) Selective angiography confirms the CT finding of a pseudoaneurysm (arrow) and identifies the feeding branch artery (arrowhead). (**D**) Angiography after a successful distal embolization shows deployment of metallic coils (arrowheads) at the branch artery and obliteration of pseudoaneurysm.

**Figure 3:**
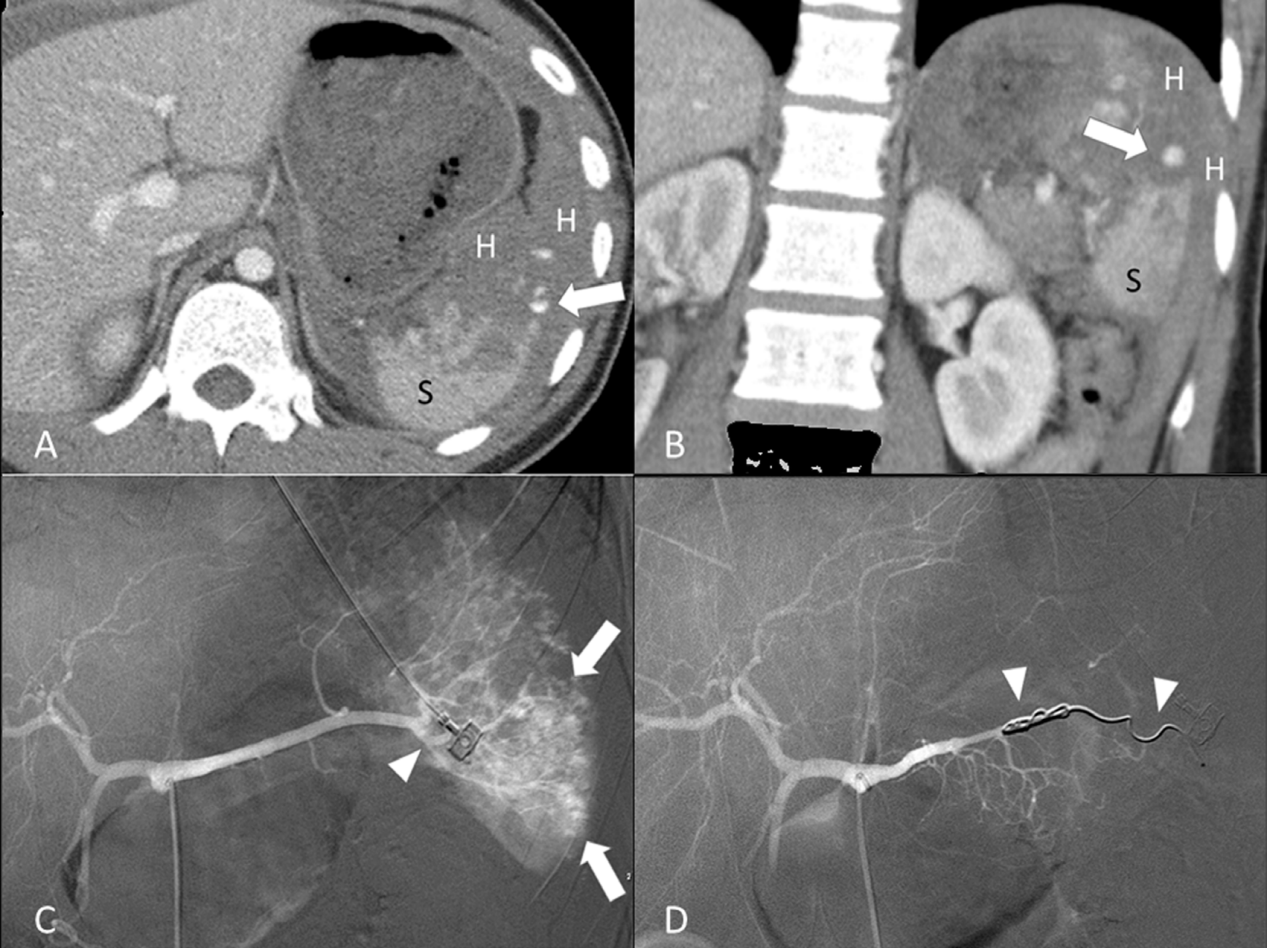
19-year-old male of grade IV splenic injury underwent combined transcatheter arterial embolization (**A**, **B**) Contrast-enhanced axial and coronal CT at portal venous phase shows a pseudoaneurysm (arrow) at the lacerated spleen (S) surrounded by hemoperitoneum (H). (**C**) Angiography discloses multiple tiny pseudoaneurysms (arrow) and the supplying branch artery (arrowhead). (**D**) Angiography after a successful combined embolization shows deployment of metallic coils (arrowheads) at the branch artery and main splenic artery distal to the dorsal pancreatic artery.

Of the 26 patients, 17 received distal TAE (Figure 2), 9 received combined TAE (Figure 3). Univariate analyses of gender, injury mechanisms, CT injury grades, number of vascular injuries on angiography (contrast medium extravasation or pseudoaneurysm) as well as age, injury severity score, pre-TAE physiological parameters and hemogram data between distal TAE group and combined TAE group were tabulated in Tables 1 and 2. None of these baseline data showed a significant difference.

**Table 1:** Comparison of baseline categorical data between distal TAE and combined TAE groups

Items		Distal TAE (*n* = 17)	Combined TAE (*n* = 9)	*p*-value
Sex				1.000
Women	(*n* = 5)	3	2	
Men	(*n* = 21)	14	7	
Injury mechanism				0.523
Motor vehicle accident	(*n* = 22)	14	8	
Pedestrian	(*n* = 2)	2	0	
Falling	(*n* = 2)	1	1	
CT injury grades				0.642
I	(*n* = 4)	2	2	
II	(*n* = 2)	2	0	
III	(*n* = 7)	5	2	
IV	(*n* = 13)	8	5	
Vascular injury on angiography				1.000
Single	(*n* = 17)	11	6	
Multiple	(*n* = 9)	6	3	

TAE = transcatheter arterial embolization.

**Table 2:** Comparison of baseline continuous data between distal TAE and combined TAE groups

Items	units	Distal TAE (*n* = 17)	Combined TAE (*n* = 9)	*p*-value
Age	(years)	44.1 ± 18.9	31.6 ± 13.6	0.120
ISS		24.1 ± 12.5	20.0 ± 14.4	0.463
Pulse rate	(beat/min)	91.4 ± 19.9	82.4 ± 8.7	0.345
Systolic pressure	(mmHg)	111.0 ± 32.9	114.8 ± 21.2	0.808
Pulse pressure	(mmHg)	46.6 ± 30.2	51.4 ± 22.9	0.666
Hemoglobin	(g/dL)	10.7 ± 3.0	11.9 ± 2.3	0.269
Hematocrit	(%)	31.6 ± 8.2	35.5 ± 6.8	0.269
Platelet count	(1000/uL)	213.8 ± 141.1	216.8 ± 82.3	0.306
International normalized ratio		1.25 ± 0.21	1.21 ± 0.17	0.804

TAE = transcatheter arterial embolization; ISS = Injury severity score.

**Figure 4:**
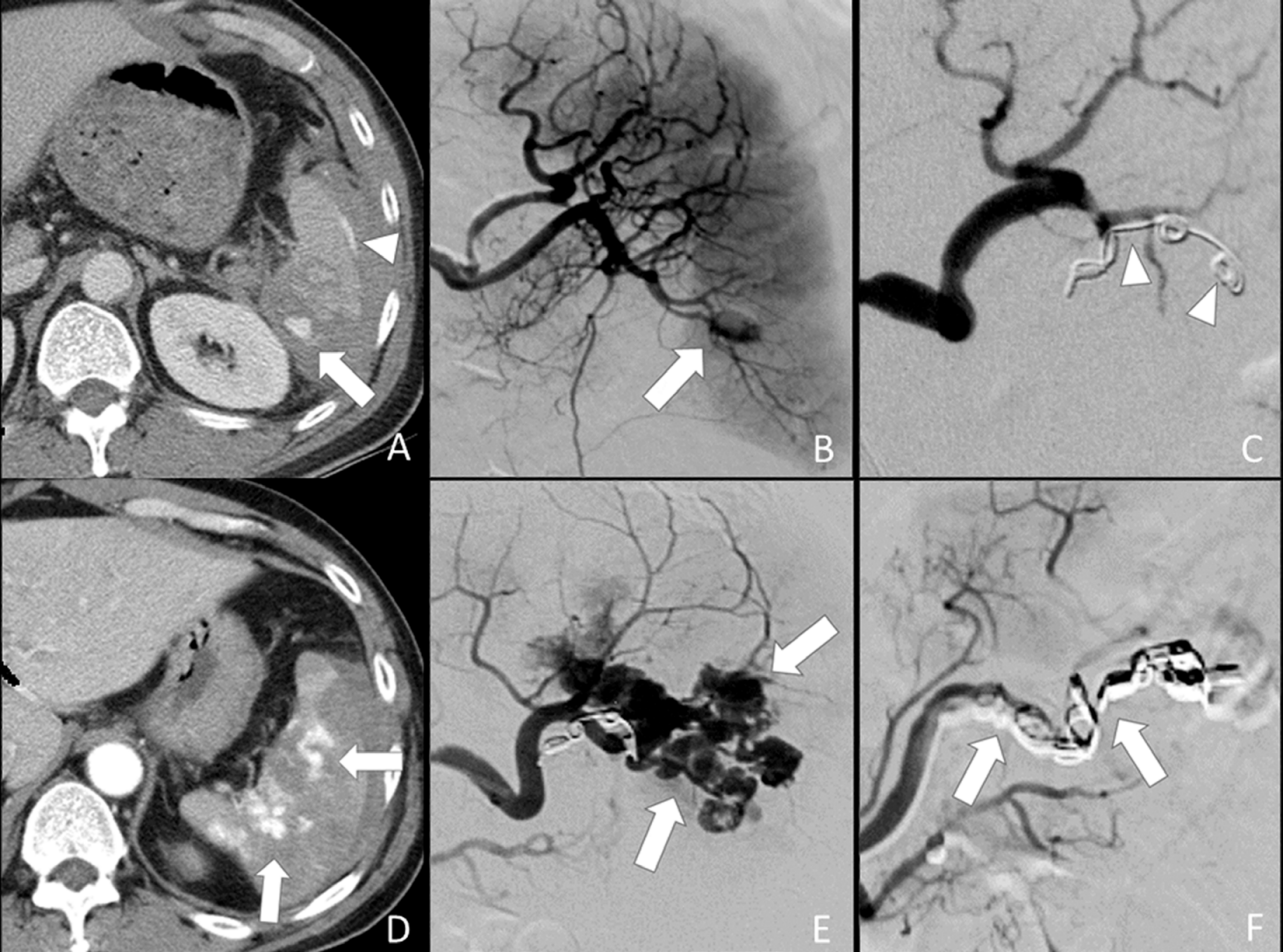
52-year-old male of grade IV splenic injury underwent combined embolization because of distal embolization failure (**A**) Contrast-enhanced axial CT shows a splenic pseudoaneurysm (arrow) and subcapsular contrast medium extravasation (arrowhead). (**B**) Selective angiography confirms the splenic pseudoaneurysm (arrow). (**C**) Metallic coils (arrowheads) were deployed at a branch artery of lower pole. Obliteration of the pseudoaneurysm is seen after distal embolization. (**D**) Arterial phase CT performed one week after embolization shows multiple new pseudoaneuryms (arrows) in the spleen, indicating a distal embolization failure. (**E**) Repeated angiography of the spleen shows multiple pseudoaneurysms (arrows). (**F**) Additional metallic coils (arrows) were deployed at the branch and main splenic artery distal to dorsal pancreatic artery for a combined embolization.

Technical success was achieved in all patients, defined by cessation of contrast medium extravasation or obliteration of pseudoaneurysm on post-embolization angiography. None of the patients in this series had significant arteriovenous fistula. We did not further separate our cases into different groups of vascular injuries for analysis because of small sample size. There was an immediate elevation of systolic blood pressure and pulse pressure after TAE in all 26 patients. These elevations reached statistical significance 24 hours after TAE (Table 3). None of them had received inotropic agents. Reduction of pulse rates was observed immediately and 24 hours after TAE but the differences were not statistically significant (Table 3). Of all 26 first follow-up CT examinations, all had a decrease of hemoperitoneum amount, 23 showed complete resolution of splenic vascular injuries. These 23 TAE was considered successful (14 distal TAE and 9 combined TAE). New splenic pseudoaneuryms that occurred at other site of the spleen were found on follow-up CT of 3 patients who had undergone distal TAE (Figure 4). These occult vascular injuries were inconspicuous on admission CT and were not embolized initially. The new pseudoaneurysms were higher in attenuation as compared to heterogeneously enhancing splenic parenchyma at arterial phase. The attenuation of pseudoaneurysm was similar to that of the aorta and splenic artery. Contrast medium in the pseudoaneurysm was washed out at the same pace as the splenic vessels during portal venous phase and equilibrium phase. These three failures with new pseudoaneurysms were confirmed and successfully treated by a second TAE in which a combined technique was employed. The risk difference of failure in distal TAE to combined TAE was 17.6% although it was not statistically significant.

**Table 3:** Wilcoxon signed rank test of elevation of physiologic parameters after TAE for blunt splenic injuries

Items	Mean difference	95% CI	*p*-value
Systolic pressure (mmHg)			
Immediate elevation	12.7 ± 30.3	(0.5, 25.0)	0.069
Elevation 24 hours later	28.2 ± 40.5	(11.9, 44.6)	0.001
Pulse pressure (mmHg)			
Immediate elevation	8.7 ± 31.8	(–4.1, 21.6)	0.322
Elevation 24 hours later	17.4 ± 31.1	(4.8, 30.0)	0.006
Pulse rate (beats per minute)			
Immediate reduction	0.6 ± 16.6	(–6.1, 7.3)	0.919
Reduction 24 hours later	5.2 ± 20.6	(–3.2, 13.5)	0.319

TAE = transcatheter arterial embolization; CI = confidence interval.

### Comparison of major post-TAE complications between distal TAE group and combined TAE group

**Figure 5:**
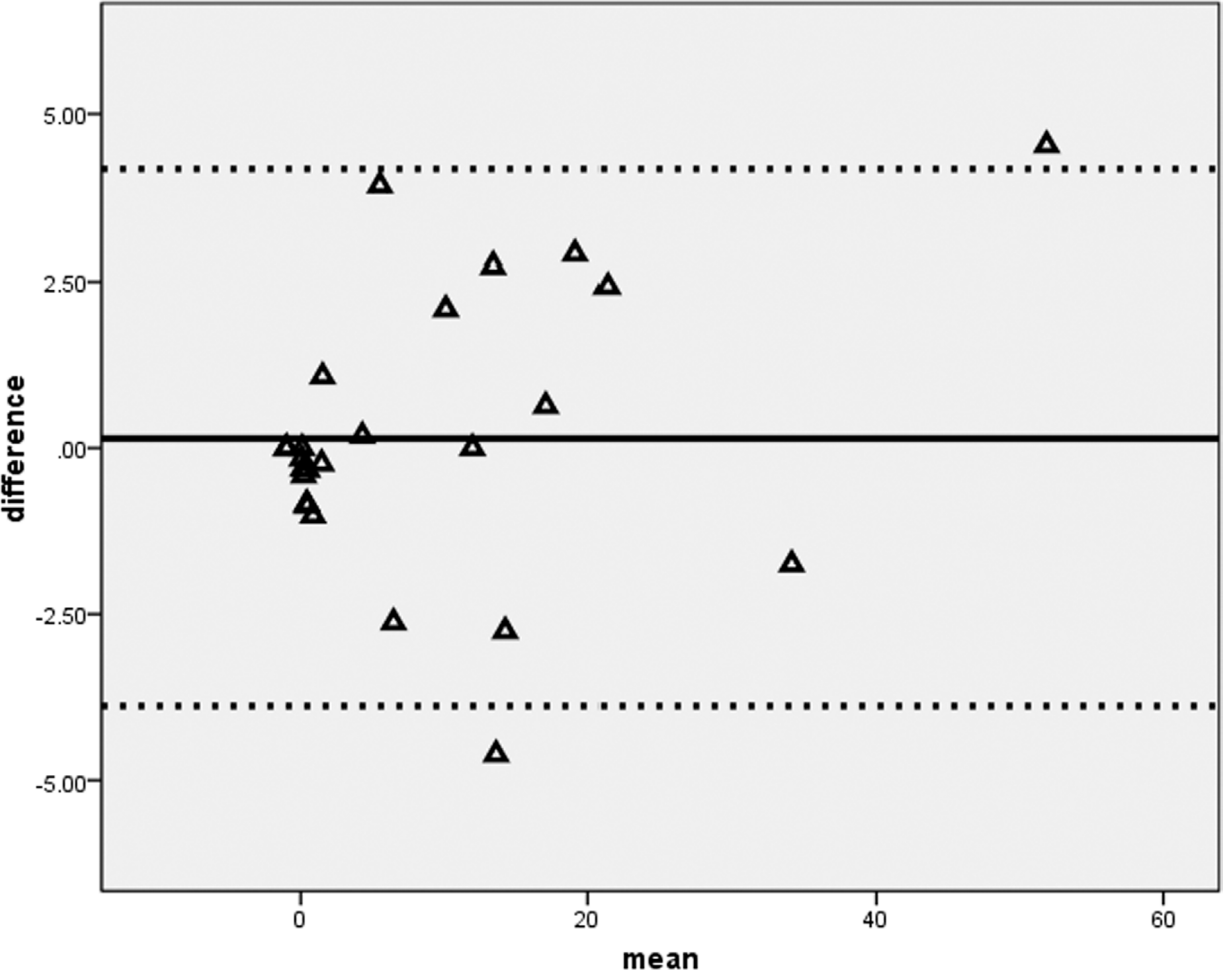
Bland-Altman difference plot of splenic infarct volume A graphical method to compare measurements differences against their average between two readers.

As tabulated in Table 4, elevation of serum amylase occurred in two patients after distal TAE. The subclinical pancreatitis was transient and the elevation of serum amylase returned to normal within one month. Although none had post-TAE elevation of amylase in combined TAE group, the difference was not significant (*p* = 0.529). None of them developed splenic abscess or massive post-TAE splenic infarct of more than 50% of spleen volume. Twenty four (92.3%) patients underwent a second follow-up CT examination. Comparison of the infarct volume percentage between distal TAE and combined TAE techniques was not statistically significant (10.9 ± 14.7% versus 6.6 ± 8.26.6 ± 8.2%, *p* = 0.848). Independent measurements of the infarct volume percentage between two readers showed good agreement with a mean difference of 0.14, standard deviation of 2.05. The upper limit was 4.2, lower limit was –3.9 (Figure 5). There was no proportion bias in the distribution on Bland-Altman plot (*p* = 0.085).

**Table 4:** Comparison of complications between distal TAE and combined TAE groups

Items	Distal TAE (*n* = 17)	Combined TAE (*n* = 9)	*p*-value
Transient elevation of serum amylase (>150 U/L)			0.529
Yes	2	0	
No	15	9	
Volume percentage of splenic infarct			0.848
	10.9 ± 14.7%	6.6 ± 8.2%	
Splenic abscess			N.A.
Yes	0	0	
No	17	9	

TAE = transcatheter arterial embolization; N.A. = non-applicable.

## DISCUSSION

Spleen TAE has been proven effective in improving the salvage rates of blunt splenic injuries although it may cause potential complications such as splenic infarct and abscess [8, 12, 13, 18]. This study showed that both the distal TAE and combined TAE techniques could elevate systolic pressure and pulse pressure as well as reduction of pulse rates of our patients indicating the efficacy of endovascular occlusion for splenic injuries [19, 20].

The concept of distal TAE technique is to occlude the branch vessel as close to the site of contrast medium extravasation as possible so that backflow from collaterals would not reconstitute the extravasation. However, distal TAE technique may cause a more complete infarct and consume a longer procedural time as compared to proximal TAE technique [8]. Moreover, distal TAE technique for high grade splenic injuries has also been reported as a predictive factor of TAE failures [10]. The rebleeding events after these superselective embolization of distal branch arteries are mostly the result of rupture of new pseudoaneurysm from the non-embolized part of the injured spleen [5].

In fact, the three embolization failures of our study were patients of distal TAE technique group. Their new pseudoaneurysms were detected at other sites of the injured spleen that were not embolized initially. These occult vascular injuries are not conspicuous at the time of vessel spasm during hypotensive state and therefore not targeted initially by distal TAE technique [21]. However, when the patients have become normotensive after successful resuscitation and TAE, the spastic vessels may reopen and lead to a life-threatening delayed splenic hemorrhage [16, 22]. One of the solutions to increase the success rate of distal TAE is to perform preemptive embolization at multiple branch arteries supplying the injured splenic parenchyma even though contrast medium extravasation or pseudoaneurysm is not detected. Obviously, this liberal TAE technique would result in a more extensive and complete splenic infarct [5, 8]. Another option is to perform proximal TAE technique which supposedly consumes a shorter procedural time and causes smaller post-TAE spleen infarcts as compared with distal TAE [5, 8]. However, it has also been reported that about 10% of proximal TAE may fail because the arterial pressure distal to the main splenic artery occlusion is highly variable and depend on the robustness of collaterals [14–16]. The reconstituted backflow from the splenic hilum would contribute to the ongoing hemorrhage despite proximal TAE [16].

Our patients who failed the distal TAE technique were subsequently treated by a second TAE in which additional coils were deployed at the main splenic artery. The repeated TAE succeeded to obliterate all pseudoaneurysms. Combined TAE technique is a combination of distal and proximal TAE. This technique not only superselectively occludes the injured branch arteries but also preemptively occludes the main splenic artery to decrease the arterial flow therefore facilitating thrombosis of occult pseudoaneurysms and reducing the risk of delayed hemorrhage. As opposed to distal TAE group, none of the combined TAE technique group had new or residual pseudoaneurysm on their follow-up CT. Our results on combined TAE technique and distal TAE technique are in agreement with the results published by Frandon J et al. [17]. We have observed the trend that distal TAE technique has a greater failure rates than the combined TAE technique. The risk difference of failure in distal TAE technique to combined TAE technique was 17.6%.

Follow-up of the injured spleen using multiphasic CT is justified because its detection of intra-parenchymal pseudoaneurysm is sensitive and specific [4, 23, 24]. Pseudoaneurysm is filled with contrast medium and appear higher in attenuation as compared to the splenic parenchyma at arterial phase. However, contrast medium in the pseudoaneurysm can be washed out at the same pace as splenic vessels and therefore it usually becomes isodense to the splenic parenchyma at portal venous phase.

Combined TAE technique is assumed to be more invasive and may result in more severe complications as compared to that of distal TAE technique. To date, there is a limited study on the potential complications of combined TAE technique. In this study, none of the patients who received combined TAE technique developed major complications such as splenic abscess, massive infarct or severe pancreatitis. The main principle of combined TAE technique is to preserve as much as possible the non-targeted part of the spleen and to avoid ischemic pancreatitis. Therefore, only the branch splenic arteries that show direct signs of injury and the main splenic artery distal to dorsal pancreatic artery are occluded.

Small sample size is the main limitation of this study. We have not investigated the immunologic complications following different spleen TAE techniques. Arterial phase CT is most accurate for detecting vascular injuries of the spleen and equilibrium phase CT is better in delineating the perfusion defect. Although multiphasic CT would cause an additional radiation dose to patients, the benefits obviously outweigh the risks.

In conclusion, combined TAE technique is effective and safe to decrease the failure rates of non-operative management of blunt splenic injuries. A larger sample study is required to verify this observation.

## MATERIALS AND METHODS

### Subjects

This prospective study was approved by Institute Review Board and a written informed consent was obtained from every patient. Within a two-year period (January 2010 to December 2011), we included all patients of blunt splenic injuries with CT findings of contrast medium extravasation. The inclusion criteria included stable hemodynamics (systolic blood pressure > 90 mmHg), clear consciousness and decision for TAE treatment by trauma team captain. We excluded patients who were less than 18 years old and allergic to contrast medium.

### TAE techniques

Patients were randomly allocated into distal TAE group and combined TAE group by a ratio of 2:1 using permuted-block randomization. Distal TAE was done by deploying metallic coils at the injured branch artery distal to the splenic hilum. Combined TAE was done by deploying metallic coils at both branch artery and main splenic artery distal to the dorsal pancreatic artery. The endpoint of branch artery TAE was total cessation of splenic contrast medium extravasation or total obliteration of pseudoaneurysm. The goal of main splenic artery TAE was to achieve an occlusion of flow distal to the site of embolization. Technical success was considered if the endpoint and goal of TAE mentioned above were evidenced on post-embolization angiography. All TAE procedures were performed by emergency radiologists who were competent at performing both diagnostic and interventional radiology at our institution. Their working experience ranged from six to twenty years.

### Clinical data

We recorded their CT injury grades in accordance with grading system of American Association for the Surgery of Trauma, mechanisms of injury, number of vascular injuries on angiography, injury severity score as well as physiology parameters and hemogram data before and after TAE. Clinical success was defined as improvement of patient's physiology parameters including blood pressure and pulse rates as well as complete resolution of vascular injuries on the first follow-up CT.

### Follow-up CT examinations after TAE

Patients underwent two follow-up multiphasic CT examinations after TAE. The first follow-up CT was performed one week after TAE, second follow-up CT was performed three months after TAE. All patients were scanned from lower lung (2 cm above diaphragm) to L4 lower endplate using a CT scanner (Somatom Sensation 16, Siemens Healthcare, Germany). A total of 120 mL of Omnipaque 350 (Iohexol, 350 mgI/ml, GE Healthcare, Ireland) was intravenously administered at a rate of 3 mL/second. The arterial-phase CT was empirically obtained at 25 second, portal venous-phase at 70 second, equilibrium-phase at 180 second respectively after initiation of intravenous contrast medium administration. All scans were obtained with 120 kVp and automatic tube current modulation technique. All thin collimation source images of 16 × 0.75 mm were reconstructed to 5 mm thickness, 5 mm interval for viewing on Picture archiving and communication system.

### Evaluation of follow-up CT examinations

All first follow-up CT examinations were read by two readers in a conference. Spleen TAE was considered clinically successful if a consensus was reached for complete resolution of splenic vascular injuries and decrease of hemoperitoneum. Splenic vascular injuries consisted of contrast medium extravasation defined as free spillage of contrast medium throughout multiphasic CT, or pseudoaneurysm defined as a confined contrast medium collection that washed out at the same pace as splenic vessels. Patients of TAE failure required additional intervention (second TAE or spleen-related surgery).

All second follow-up CT examinations were evaluated for splenic infarct. The equilibrium-phase images obtained were loaded to a three dimensional visualization and analysis software (Amira 3D, version 4.1). The structures of interest (infarcted spleen and viable spleen) on every contiguous axial scans were marked freehand around the margin using an electronic cursor by two readers independently. The infarcted spleen was defined as an area of decreased enhancement as compared to the viable spleen.

### TAE-related complications

Post-TAE infarcted volume of more than 50% of the spleen, development of splenic abscess or elevation of serum amylase level (> 150 U/L) after TAE as compared to the serum amylase on admission were all recorded as major post-TAE complications.

### Statistics

Chi square test or Fisher's exact test was used to compare categorical data. Mann-Whitney test and Wilcoxon signed ranks test were used for comparisons of continuous data. Bland-Altman plot method was used to test the agreement between two readers regarding measurements of the infarcted spleen and viable spleen volumes. A 2-tailed *p*-value of < 0.05 was considered statistically significant.

## Abbreviations

TAE: transcatheter arterial embolization.
